# Nonstructural proteins 2B and 4A of Tembusu virus induce complete autophagy to promote viral multiplication in vitro

**DOI:** 10.1186/s13567-023-01152-2

**Published:** 2023-03-14

**Authors:** Wangyang Tan, Senzhao Zhang, Yu He, Zhen Wu, Mingshu Wang, Renyong Jia, Dekang Zhu, Mafeng Liu, Xinxin Zhao, Qiao Yang, Ying Wu, Shaqiu Zhang, Juan Huang, Sai Mao, Xumin Ou, Qun Gao, Di Sun, Bin Tian, Shun Chen, Anchun Cheng

**Affiliations:** 1 Institute of Preventive Veterinary Medicine, Sichuan Agricultural University, Chengdu, 611130 Sichuan China; 2 Research Center of Avian Disease, College of Veterinary Medicine, Sichuan Agricultural University, Chengdu, 611130 Sichuan China; 3 Key Laboratory of Animal Disease and Human Health of Sichuan Province, Sichuan Agricultural University, Chengdu, 611130 Sichuan China

**Keywords:** Tembusu virus, autophagy, nonstructural protein 2B, nonstructural protein 4A, p62

## Abstract

Tembusu virus (TMUV) is an emerging flavivirus that has broken out in different regions of China. TMUV infection has been reported to induce autophagy in duck embryo fibroblast cells. However, the molecular mechanisms underlying this autophagy induction remain unclear. Here, we explored the interactions between autophagy and TMUV and the effects of the structural and nonstructural proteins of TMUV on autophagy in vitro. Among our results, TMUV infection enhanced autophagy to facilitate viral replication in HEK293T cells. After pharmacologically inducing autophagy with rapamycin (Rapa), the replication of TMUV increased by a maximum of 14-fold compared with the control group. To determine which TMUV protein primarily induced autophagy, cells were transfected with two structural proteins and seven nonstructural proteins of TMUV. Western blotting showed that nonstructural proteins 2B (NS2B) and 4 A (NS4A) of TMUV significantly induced the conversion of microtubule-associated protein 1 light chain 3 (LC3) from LC3-I to LC3-II in HEK293T cells. In addition, through immunofluorescence assays, we found that NS2B and NS4A significantly increased the punctate fluorescence of GFP-LC3-II. Furthermore, we found that both NS2B and NS4A interacted with polyubiquitin-binding protein sequestosome 1 (SQSTM1/p62) in a coimmunoprecipitation assay. Moreover, the autophagic degradation of p62 and LC3 mediated by NS2B or NS4A was inhibited by treatment with the autophagic flux inhibitor chloroquine (CQ). These results confirmed the vital effects of NS2B and NS4A in TMUV-induced complete autophagy and clarified the importance of complete autophagy for viral replication, providing novel insight into the relationship between TMUV and autophagy.

**Supplementary Information:**

The online version contains supplementary material available at 10.1186/s13567-023-01152-2.

## Introduction

Tembusu virus (TMUV) is an emerging flavivirus belonging to the genus *Flavivirus*, family *Flaviviridae*, that can cause acute egg-drop syndrome in egg-laying ducks, leading to great economic loss to the poultry industry in China [1]. The *Flavivirus* genus also includes dengue virus (DENV), Zika virus (ZIKV), West Nile virus (WNV), yellow fever virus (YFV) and Japanese encephalitis virus (JEV), which seriously endanger human life and health [2]. TMUV has a wide range of hosts, including ducks, chickens, geese, mice and even humans, which makes the virus a potential threat to the public health of human beings [3–5]. Similar to other flaviviruses, TMUV has an ~11kb positive-strand RNA genome, composed of a single open reading frame (ORF) that encodes three structural proteins (C, prM, E) and seven nonstructural (NS) proteins (NS1, NS2A, NS2B, NS3, NS4A, NS4B, NS5) [6]. These proteins participate in the generation of viral progeny, the host antiviral immune response, the inflammatory response, endoplasmic reticulum remodeling, and autophagy, among other processes [6, 7].

Autophagy is a dynamic process involving the rearrangement of subcellular membranes, during which the cytoplasm and organelles are sequestered in lysosomes or vacuoles for degradation to allow recycling of the sequestered cargos [8, 9]. Autophagy is activated by various intracellular and extracellular stresses, including nutrient deficiency, organelle damage, accumulation of unfolded proteins, accumulation of lipids, and viral infection [10, 11]. Complete autophagy involves three major processes, the formation of autophagosomes and simultaneous capture of cytoplasmic cargos, the fusion of autophagosomes and lysosomes to form autolysosomes, and the degradation and turnover of cargos in autolysosomes [6].The microtubule-associated protein 1 light chain 3 (LC3), a member of the Atg8-family protein, is involved in autophagosome formation [11], where LC3 is converted from LC3-I to LC3-II, which is a marker for monitoring enhanced autophagy [12]. Sequestosome 1 (SQSTM1/p62) serves as a link between LC3 and ubiquitinated substrates [13] that become incorporated into the completed autophagosome and are degraded in autolysosomes, thus serving as a hallmark of autophagic degradation.

Although autophagy is supposed to be activated as an antiviral response during viral infection, a growing number of studies have found that flaviviruses hijack autophagy to regulate their life cycle and promote their replication and propagation [11, 14, 15]. In addition, there are many protein–protein interactions between flaviviruses and autophagy, which regulate not only the replication of the virus but also the stress response of the host caused by virus infection [6, 16]. For example, DENV NS1 interacts with Beclin-1 to activate autophagy and prevent apoptosis in the early stages of infection, which promotes viral replication [17]. WNV C protein interacts with adenosine 5′-monophosphate-activated protein kinase (AMPK), promoting the degradation of AMPK to inhibit autophagy, which contributes to the development of neurological disease [18]. In addition, ZIKV NS5 interacts with the host protein Ajuba to inhibit mitophagy, which promotes the production of early pro-inflammatory chemokines and viral propagation [19]. Although autophagy is involved in the replication of various flaviviruses, the role of autophagy as a proviral or antiviral function in flavivirus replication is complex and has been less fully elucidated [6, 14]. Our recent studies showed that TMUV infection can induce autophagy in duck embryo fibroblasts (DEFs), which inhibits the TANK-*binding kinase 1 (TBK1)-mediated host antiviral immune response to promote viral replication [12]. Previous studies have shown that various flavivirus proteins play important roles in autophagy and virus replication [17, 20, 21], however, the role of TMUV proteins in autophagy is still unclear.

In this study, using Western blotting and immunofluorescence assays, we found that TMUV infection promoted the conversion of LC3-I to LC3-II in HEK293T cells and the formation of GFP-LC3 punctate fluorescence in BHK-21 cells, which verified that TMUV infection can induce autophagy in mammalian cell lines. Through Western blotting and RT-qPCR assays, we found that rapamycin (Rapa), a complete autophagy inducer, promoted TMUV replication in HEK293T cells upon TMUV infection. Further study showed that NS2B and NS4A are two key proteins that mediate TMUV-induced autophagy. In addition, via immunoprecipitation and immunofluorescence assays, we found that NS2B and NS4A interact with p62 to enhance autophagic flux, which suggests that NS2B and NS4A induce complete autophagy to promote TMUV replication. These results suggest a crucial role of NS2B and NS4A in TMUV-induced complete autophagy and clarify the importance of complete autophagy for viral replication, broadening the current understanding of the relationship between TMUV and autophagy.

## Materials and methods

### Virus and plasmids

The CQW1 strain and the eukaryotic expression vectors pCAGGS, pCAGGS-P62-Flag, GFP-LC3 and GFP-RFP-LC3 (ptf-LC3) were all preserved and provided by the Poultry Disease Control and Research Center of Sichuan Agricultural University. The genes C-HA, prM-HA, NS1-HA, NS2A-HA, NS2B-HA, NS3-HA, NS2B3-HA, NS4A-HA, NS4B-HA and NS5-HA were amplified from the pACNR CQW1-Intron plasmid [22] using the primers in Table 1. Then the amplified fragments were cloned into the pCAGGS vector to construct the eukaryotic recombinant plasmids pCAGGS-C-HA, pCAGGS-prM-HA, pCAGGS-NS1-HA, pCAGGS-NS2A-HA, pCAGGS-NS2B-HA, pCAGGS-NS3-HA, pCAGGS-NS2B3-HA, pCAGGS-NS4A-HA, pCAGGS-NS4B-HA and pCAGGS-NS5-HA.

**Table 1:** **Primers used in this study**

Name	Sequence(5’→3’)	Purpose
pCAGGS-C-HA-F	CATCATTTTGGCAAAGCCACCATGTCTAACAAAAAACCAGGA	Gene cloning
pCAGGS-C-HA-R	TTGGCAGAGGGAAAACTAAGCGTAATCTGGAACATCGTATGGGTACCGACGTTTCGCCTTCCG	
pCAGGS-prM-HA-F	CATCATTTTGGCAAAGCCACCATGGGGGGGAGTTGCTCTTGG	
pCAGGS-prM-HA-R	TTGGCAGAGGGAAAACTAAGCGTAATCTGGAACATCGTATGGGTAGCTGTACGCTGGGGCAAT	
pCAGGS-NS1-HA-F	CATCATTTTGGCAAAGCCACCATGGGCCTGAATGCAAGGGAC	
pCAGGS-NS1-HA-R	TTGGCAGAGGGAAAACTAAGCGTAATCTGGAACATCGTATGGGTAAGCCATGACCTTTGATTT	
pCAGGS-NS2A-HA-F	CATCATTTTGGCAAAGCCACCATGTTTCAAGGGGGTGGCATG	
pCAGGS-NS2A-HA-R	TTGGCAGAGGGAAAACTAAGCGTAATCTGGAACATCGTATGGGTATCTCCGTGTCACTGGCTT	
pCAGGS-NS2B-HA-F	CATCATTTTGGCAAAGCCACCATGGGGTGGCCAGTCAGTGAG	
pCAGGS-NS2B-HA-R	TTGGCAGAGGGAAAACTAAGCGTAATCTGGAACATCGTATGGGTATCGTTGTTTTGCCTTAGT	
pCAGGS-NS3-HA-F	CATCATTTTGGCAAAGCCACCATGGGAGGAGTCATCTGGGAT	
pCAGGS-NS3-HA-R	TTGGCAGAGGGAAAACTAAGCGTAATCTGGAACATCGTATGGGTATCTCTTTCCACTCGCAAA	
pCAGGS-NS2B3-HA-F	CATCATTTTGGCAAAGCCACCATGGGGTGGCCAGTCAGTGAG	
pCAGGS-NS2B3-HA-R	TTGGCAGAGGGAAAACTAAGCGTAATCTGGAACATCGTATGGGTATCTCTTTCCACTCGCAAA	
pCAGGS-NS4A-HA-F	CATCATTTTGGCAAAGCCACCATGTCAGCGATAGGGATCCTT	
pCAGGS-NS4A-HA-R	TTGGCAGAGGGAAAACTAAGCGTAATCTGGAACATCGTATGGGTATCTCTGTCTCTCTGGTTC	
pCAGGS-NS4B-HA-F	CATCATTTTGGCAAAGCCACCATGTCACAGACGGATAGTCAC	
pCAGGS-NS4B-HA-R	TTGGCAGAGGGAAAACTAAGCGTAATCTGGAACATCGTATGGGTATCGACGCAAGGTCCCTGC	
pCAGGS-NS5-HA-F	CATCATTTTGGCAAAGCCACCATGGGAGGAGGAACTGGCAGA	
pCAGGS-NS5-HA-R	TTGGCAGAGGGAAAACTAAGCGTAATCTGGAACATCGTATGGGTACAAAACACCTTCACTCCA	
TMUV-E-F	AATGGCTGTGGCTTGTTTGG	qPCR
TMUV-E-R	GGGCGTTATCACGAATCTA	
GAPDH-F	GGAGCGAGATCCCTCCAAAAT	
GAPDH-R	GGCTGTTGTCATACTTCTCATGG	

### Reagents and antibodies

Rapamycin (Rapa) (HY-10,219), chloroquine (CQ) (HY-17,589) and 3-Methyladenine (3-MA) (HY-19,312) were purchased from MedChemExpress (MCE, Shanghai, China).

Anti-LC3B antibody (T55992) and anti-HA-tag antibody (M20003) were purchased from Abmart (Shanghai, China). Anti-p62 antibody was purchased from Abcam (ab109012, Shanghai, China). Anti-β-actin antibody (HC201-01) and anti-Flag-tag antibody (HT201-02) were purchased from TransGen Biotech (Beijing, China). A polyclonal antibody against the TMUV-NS3 protein was prepared in our laboratory. Horseradish peroxidase (HRP)-conjugated anti-rabbit immunoglobulin (IgG) antibody (1 706 515) and HRP-conjugated anti-mouse IgG antibody (1 706 516) were purchased from Bio-Rad (USA).

### Cell culture, transfection, and viral infection

The baby hamster kidney fibroblast (BHK-21) cell line was cultured in Dulbecco’s modified Eagle’s medium (DMEM) (BasalMedia, Shanghai, China) supplemented with 10% fetal bovine serum (FBS) (Gibco, New York, USA) and incubated at 37 ℃ with 5% CO_2_. The human embryonic kidney 293T (HEK293T) cell line was cultured in RPMI 1640 basic medium (Gibco, Beijing, China) supplemented with 10% FBS (Gibco, New York, USA) and incubated at 37 ℃ with 5% CO_2_. Cell transfection was performed using Lipofectamine 3000 Reagent (Invitrogen, 2 423 116) according to the procedures recommended by the manufacturer. For viral infection, cells were infected with rCQW1 at different 50% tissue culture infective doses (TCID_50_) followed by incubation at 37 ℃ for 2h to ensure viral adsorption. After being washed 2 times with phosphate-buffered saline (PBS), the cells were subsequently cultured in fresh DMEM or RPMI-1640 with 2% FBS and 1% penicillin/streptomycin at 37 ℃ for different times until being harvested.

### Western blotting analysis

Cells were washed twice with cold PBS and subsequently incubated with RIPA lysis buffer (89,901, Thermo Fisher Scientific, USA) containing 1% phenylmethanesulfonyl fluoride (PMSF) (ST506, Beyotime, Shanghai, China) at 4 ℃ for 20min. The lysates were clarified by centrifugation at 12 000 rpm for 10 min at 4 ℃. Then, the protein samples were boiled for 10 min. Equal amounts of protein samples were separated by 12% or 15% SDS–PAGE. The separated proteins were electrotransferred onto polyvinylidene difluoride (PVDF) membranes (1,620,177, Bio-Rad, USA) using a Bio-Rad semidry transfer system. The membranes were blocked with 5% nonfat milk in Tris-buffered saline containing 0.1% Tween 20 (TBST) for 2~3 h at room temperature and subsequently incubated with specific primary antibodies (1:1000 for LC3B, 1:2000 for p62 and 1:3000 for all others) at 4 ℃ overnight. After three washes with TBST, the membranes were incubated with appropriate HRP-conjugated secondary antibodies (1:3000) for 1~2 h at room temperature and detected with Clarity Western ECL Substrate (Bio-Rad, USA) in a Bio-Rad imager. Bands were analyzed with Image J software.

### Immunofluorescence assay

Subconfluent cells in 12-well plates were transfected with the indicated plasmids. Cells were fixed with 4% paraformaldehyde at 4 ℃ overnight and then permeabilized with 0.3% Triton X-100 in PBS at 4 ℃ for 1 h. Nuclei were stained with 4’,6-diamidino-2-phenylindole (DAPI) (Solarbio, China) for 20 min at room temperature. Then images were acquired with a fluorescence microscope (BX53 + andorDU888, OLYMPUS, Japan).

### Reverse transcription and quantitative real-time PCR (RT–qPCR) analysis

Total RNA was extracted from cells using RNAiso Plus reagent (9109, Takara, Japan) according to the manufacturer’s instructions, and 1 µg of total RNA was reverse-transcribed using HiScript III RT SuperMix (R323-01, Vazyme, Nanjing, China). RT–qPCR was performed using 2 × Taq SYBR Green qPCR Premix (Innovagene, Changsha, China) with the Bio-Rad CFX96 Real-Time Detection System (Bio-Rad, Hercules, CA, USA). The Ct values were normalized to GAPDH gene, and the expression of each gene was represented as 2^(−ΔΔCt)^ relative to control. The primer sequences used for qRT–PCR were listed in Table 1.

### Coimmunoprecipitation assay

Cells were washed twice with cold PBS and subsequently incubated with IP lysis buffer (87788, Thermo Fisher Scientific, USA) containing 1% PMSF at 4 ℃ for 20 min. The lysates were centrifuged and incubated with anti-Flag-tag antibody overnight followed by incubation with magnetic agarose beads (1614023, Bio-Rad, USA) for 5 h. The beads were washed 3 times using cold TBST and boiled in sample buffer for 10 min. The precipitated proteins within the eluted fractions were analyzed via Western blotting as described above.

### Statistical analysis

All experiments were repeated at least three times independently. The data were analyzed with Student’s *t* test and one-way analysis of variance (ANOVA) using GraphPad Prism (version 8.4.2). Data are presented as the means ± standard deviations (SD). For significant differences, a *P* value < 0.05 was considered statistically significant.

## Results

### TMUV infection induces autophagy

**Figure 1:**
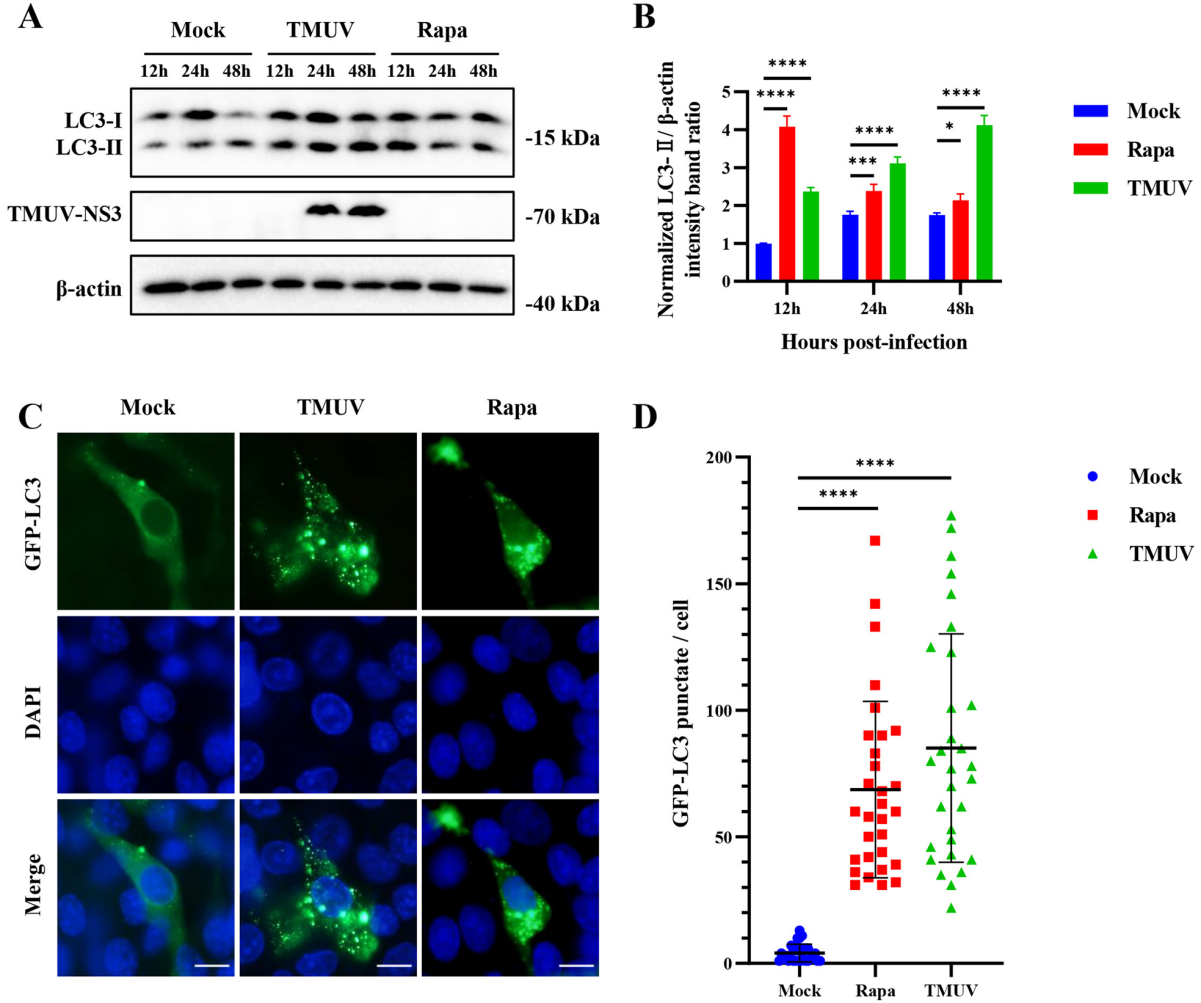
**TMUV infection induces autophagy**. **A** HEK293T cells were infected with TMUV or treated with Rapa (200 Nm) for 12, 24, and 48 h. Samples were harvested for Western blotting analysis of the proteins LC3, TMUV-NS3, and β-actin. **B** Normalized LC3-II/β-actin intensity band ratio from the data in **A**. **C** BHK-21 cells were transfected with pEGFP-LC3 and infected with TMUV or treated with Rapa (200 nM). Cells were fixed and imaged for GFP fluorescence after transfection for 48 h. Scale bar, 10 μm. **D** Number of GFP-LC3 puncta per cell. The GFP-LC3 puncta were counted in 30 cells per group. Student’s *t* test was performed to determine statistical significance (*, *P* < 0.05; **, *P* < 0.01; ***, *P* < 0.001; ****, *P* < 0.0001).

We previously demonstrated that TMUV infection can induce autophagy in DEFs [12]. To determine whether TMUV infection can induce autophagy in other cell lines, HEK293T and BHK-21 cells were infected with TMUV at a dose of 10^5^ TCID_50_. Rapa is a TOR complex 1 inhibitor that can induce complete autophagy [23] and was utilized as a positive control in this study. As shown in Figures 1A and B, TMUV infection significantly increased the conversion of LC3-I to LC3-II compared to the mock-infection group, resulting in an approximately 2-foldincrease in the amount of LC3-II. And the amount of LC3-II also increased with the extension of infection time. To further verify the effect of TMUV on the formation of autophagosomes, BHK-21 cells were transfected with GFP-LC3 plasmids to observe the formation of punctate GFP-LC3 fluorescence. During the formation of autophagosomes, diffuse GFP- LC3-I is converted to punctate GFP-LC3-II. As expected, TMUV infection resulted in a significant increase in punctate GFP-LC3-II fluorescence (Figures 1C and D). All these data suggest that TMUV infection can induce autophagy in vitro.

### Autophagy promotes TMUV replication

**Figure 2:**
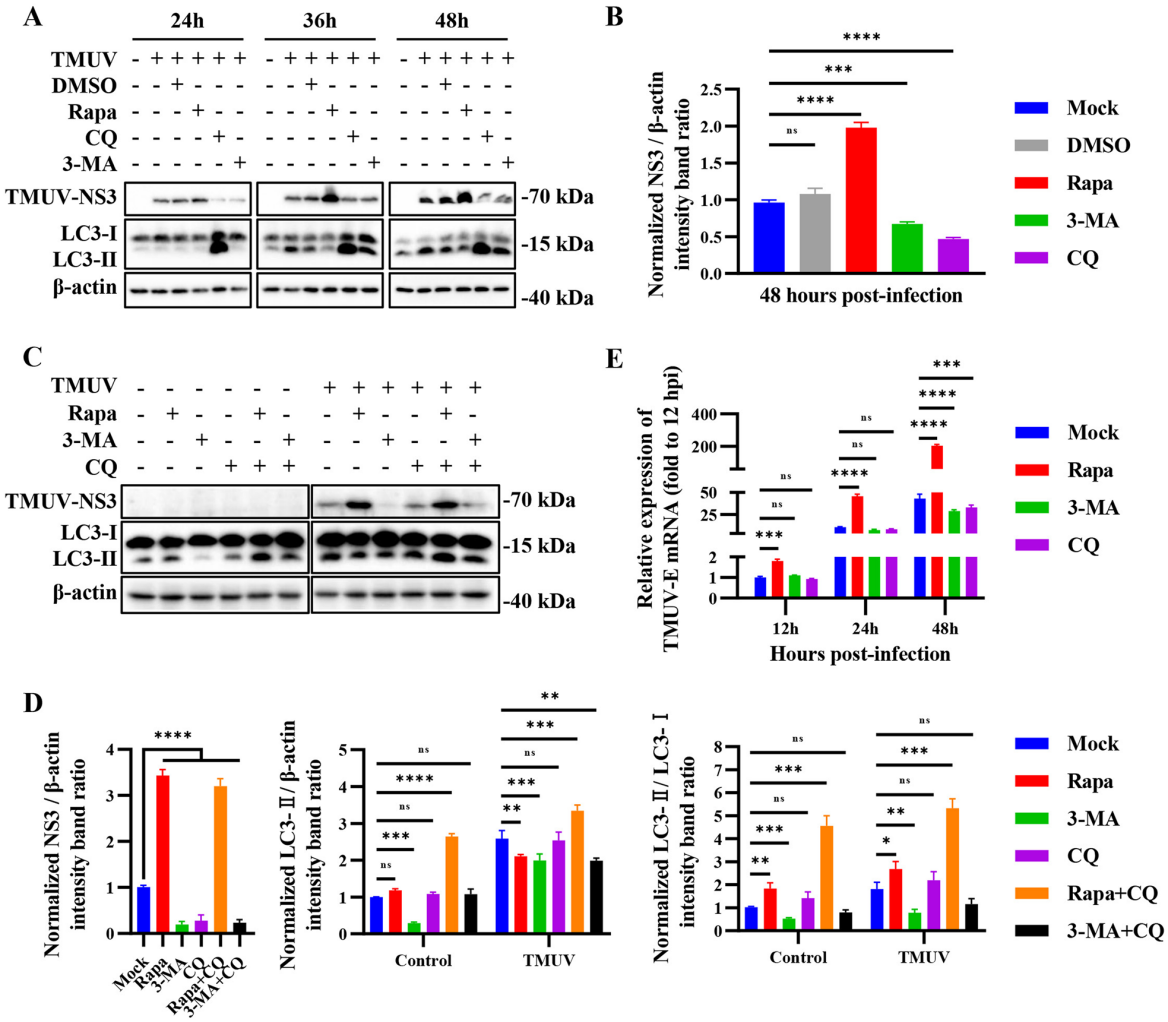
**Autophagy promotes TMUV replication. A** HEK293T cells were treated with RAPA (200 nM), CQ (40 µM) or 3-MA (5 mM) for 6 h to induce or inhibit autophagy, cells treated with equal volume DMSO were used as the negative control and untreated cells were used as the mock control. Then, the cells were infected with TMUV for 12, 24, and 48 h. Cell lysates were harvested at the indicated times and analyzed via Western blotting with anti-LC3, anti-NS3 and anti-β-actin antibodies. **B** Normalized NS3/β-actin intensity band ratio from the data in **A**. **C** HEK293T cells were treated with RAPA (200 nM), CQ (40 µM) or 3-MA (5 mM) for 6 h alone or in combination, and then, the cells were infected with TMUV for 48 h. Cell lysates were harvested and analyzed via Western blotting with anti-LC3, anti-NS3 and anti-β-actin antibodies. **D** Normalized NS3/β-actin, LC3-II/β-actin, and LC3-II/LC3-I intensity band ratios from the data in **C**. **E** HEK293T cells were treated with RAPA (200 nM), CQ (40 µM) or 3-MA (5 mM) for 6 h to induce or inhibit autophagy, and then, the cells were infected with TMUV for 12, 24, and 48 h. Cell lysates were harvested at the indicated times for qPCR to test the relative expression of the E gene. The expression of the E gene in the mock group was set as 1. Student’s *t* test and one-way ANOVA were performed to determine statistical significance (*, *P* < 0.05; **, *P* < 0.01; ***, *P* < 0.001; ****, *P* < 0.0001).

To further study the role of autophagy in TMUV replication, we detected the level of the viral protein NS3 and virus titers in the presence or absence of Rapa, 3-methyladenine (3-MA) and chloroquine (CQ) upon TMUV infection. 3-MA, an inhibitor of class III phosphatidylinositol 3-kinase, resulting in autophagy inhibition, was used to inhibit the formation of autophagosomes [24]. CQ, which can raise the lysosomal pH and ultimately inhibit the fusion between autophagosomes and lysosomes, was used to prevent the maturation of autophagosomes into autolysosomes [25]. To evaluate viral multiplication in pharmaceutical-treated cells, the production of TMUV NS3 was examined via immunoblotting at the indicated time points. As shown in Figures 2A and B, the production of NS3 was significantly enhanced by Rapa at 36 and 48 h post-infection (hpi) and inhibited by 3-MA and CQ at 24 and 48 hpi. In particular, the production of NS3 was increased approximately 2-fold by Rapa at 48 hpi, which suggests that autophagy promotes viral replication. However, to our surprise, the amount of LC3-II in Rapa-treated cells was not greater than that in mock-treated cells (Figure 2A). This may be indicative of higher basal autophagic levels and stronger autophagic flux activity enhanced by TMUV infection, leading to increased degradation of LC3-II. To confirm our suspicions, we performed Western blotting of HEK293T cells treated with Rapa, 3-MA or CQ alone or in combination. Consistent with our hypothesis, although CQ treatment didn’t enhance the production of LC3-II in mock-infected cells and TMUV-infected cells, cotreatment with Rapa and CQ reversed the degradation of LC3-II, resulting in higher amounts of LC3-II and higher LC3-II/LC3-I ratios than those in mock-treated and Rapa-treated cells among both mock-infected cells and TMUV-infected cells, which indicated strong autophagic flux activity in Rapa-treated cells (Figures 2C and D). To verify the proviral effect of autophagy during TMUV infection, we utilized RT–qPCR to evaluate multiplication kinetics in HEK293T cells treated with Rapa, 3-MA or CQ. Consistent with the Western blotting results, Rapa promoted TMUV replication, while CQ and 3-MA significantly inhibited TMUV replication (Figure 2E). These data point to a proviral role for autophagy in TMUV replication.

### TMUV NS2B and NS4A induce autophagy

**Figure 3:**
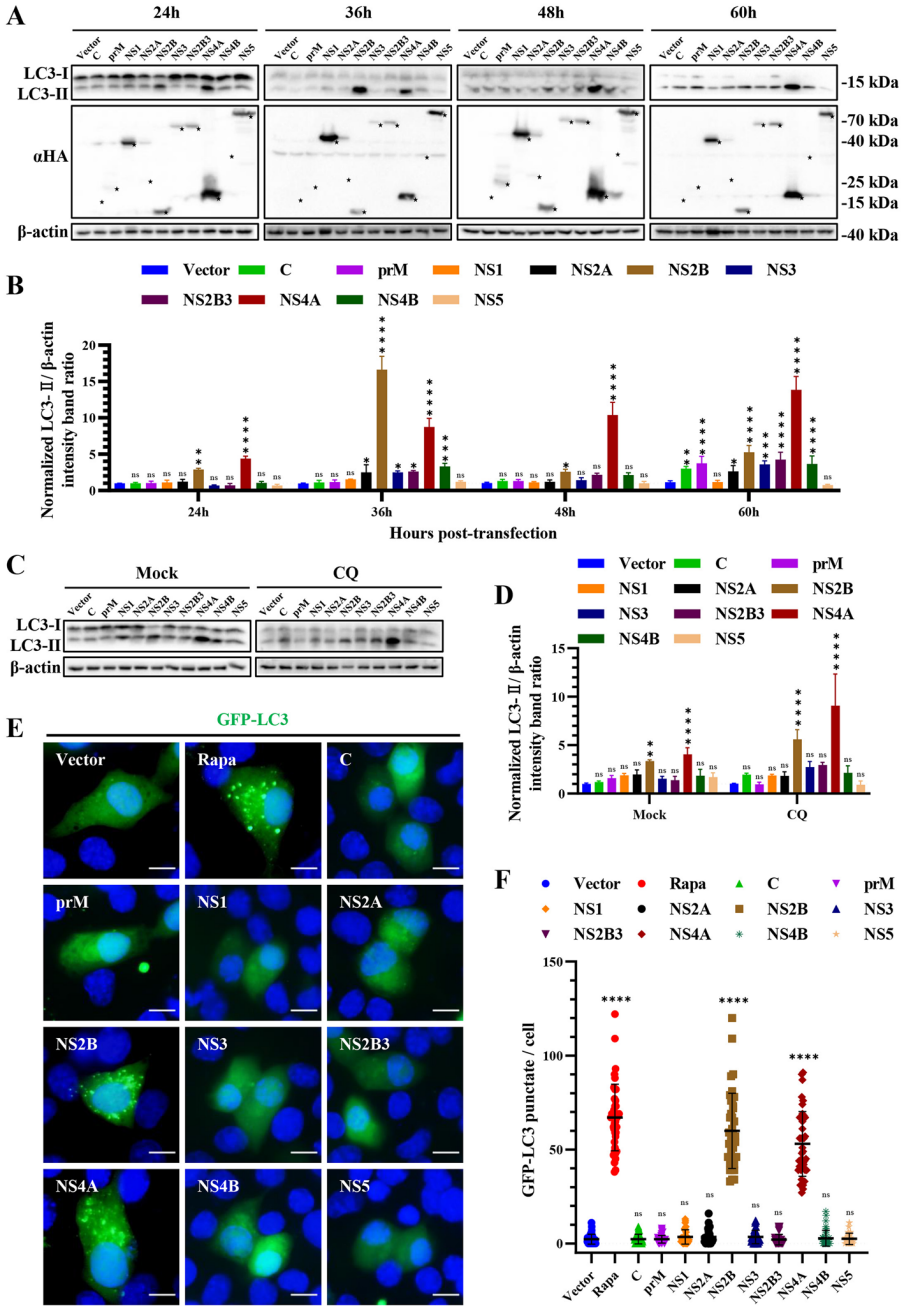
**TMUV NS2B and NS4A induce autophagy**. **A** HEK293T cells were transfected with pCAGGS empty vector and 10 protein plasmids for 24, 36, 48, and 60 h. Samples were harvested for Western blotting analysis and immunoblotted for the proteins LC3, HA, and β-actin. Bands labeled by “★” indicated the expressed specific viral proteins. **B** Normalized LC3-II/β-actin intensity band ratio from the data in **A**. **(C)** HEK293T cells were transfected with pCAGGS empty vector and 10 protein plasmids for 30 h. To inhibit the autophagic degradation of LC3, cells were treated with CQ (40 µM) for 24 h and then harvested. **D** Normalized LC3-II/β-actin intensity band ratio from the data in **C**. **E** BHK-21 cells were co-transfected with pEGFP-LC3 and ten TMUV protein plasmids. Cells co-transfected with pEGFP-LC3 and pCAGGS vector plasmids were used as the negative controls, and the cells transfected with pEGFP-LC3 and treated with Rapa (200 nM) were as the positive controls. Cells were fixed and imaged for GFP fluorescence after transfection for 36 h. Scale bar, 10 μm. **F** Number of the GFP-LC3 puncta per cell. The GFP-LC3 puncta were counted in 40 cells per group. One-way ANOVA was performed to determine statistical significance (*, *P* < 0.05; **, *P* < 0.01; ***, *P* < 0.001; ****, *P* < 0.0001).

Since TMUV infection induced autophagy in HEK293T cells, we further examined which protein encoded by TMUV could induce autophagy. Ten proteins (C, prM, NS1, NS2A, NS2B, NS3, NS2B3, NS4A, NS4B, NS5) encoded by TMUV with an HA tag were overexpressed in HEK293T cells after being cloned into the pCAGGS vector. The empty pCAGGS vector was used as a negative control. Through Western blotting, we found that all HA-tagged TMUV proteins were expressed well in transfected HEK293T cells (Figure 3A and Additional file 1). Interestingly, the expression of NS2B and NS4A resulted in a significant increase in LC3-I to LC3-II conversion from 24 to 60 hpi, respectively resulting in a maximum 18-fold and 15-fold increases in the amount of LC3-II (Figures 3A and B). To exclude the effect of autophagic degradation on this process, CQ was used to verify the results. As shown in Figures 3C and D, the expression of NS2B and NS4A indeed led to a significant increase in LC3-I to LC3-II conversion in the presence or absence of CQ. Then, BHK-21 cells were co-transfected with the ten proteins and GFP-LC3 to study their effect on the formation of autophagosomes. Consistent with the Western blotting results, the expression of NS2B and NS4A significantly promoted the formation of punctate GFP-LC3 fluorescence in BHK-21 cells, indicating that NS2B and NS4A can induce the formation of autophagosomes (Figures 3E and F). Overall, these results suggest that NS2B and NS4A are two vital viral proteins in TMUV-induced autophagy.

### TMUV NS2B and NS4A promote the degradation of p62

In general, autophagy is a dynamic and continuous process in which the generation of autophagosomes is accompanied by the simultaneous degradation of autophagosomes and autophagic cargo in cells [26]. p62, an autophagy receptor, links ubiquitinated proteins to LC3, which is vital for the degradation of autophagic cargo [27]. Together with LC3 and other autophagic cargos, p62 is degraded by lysosomal hydrolases in autolysosomes [15]. Therefore, one of the hallmarks to determine autophagic flux is whether p62 and LC3 degradation occurs.

**Figure 4:**
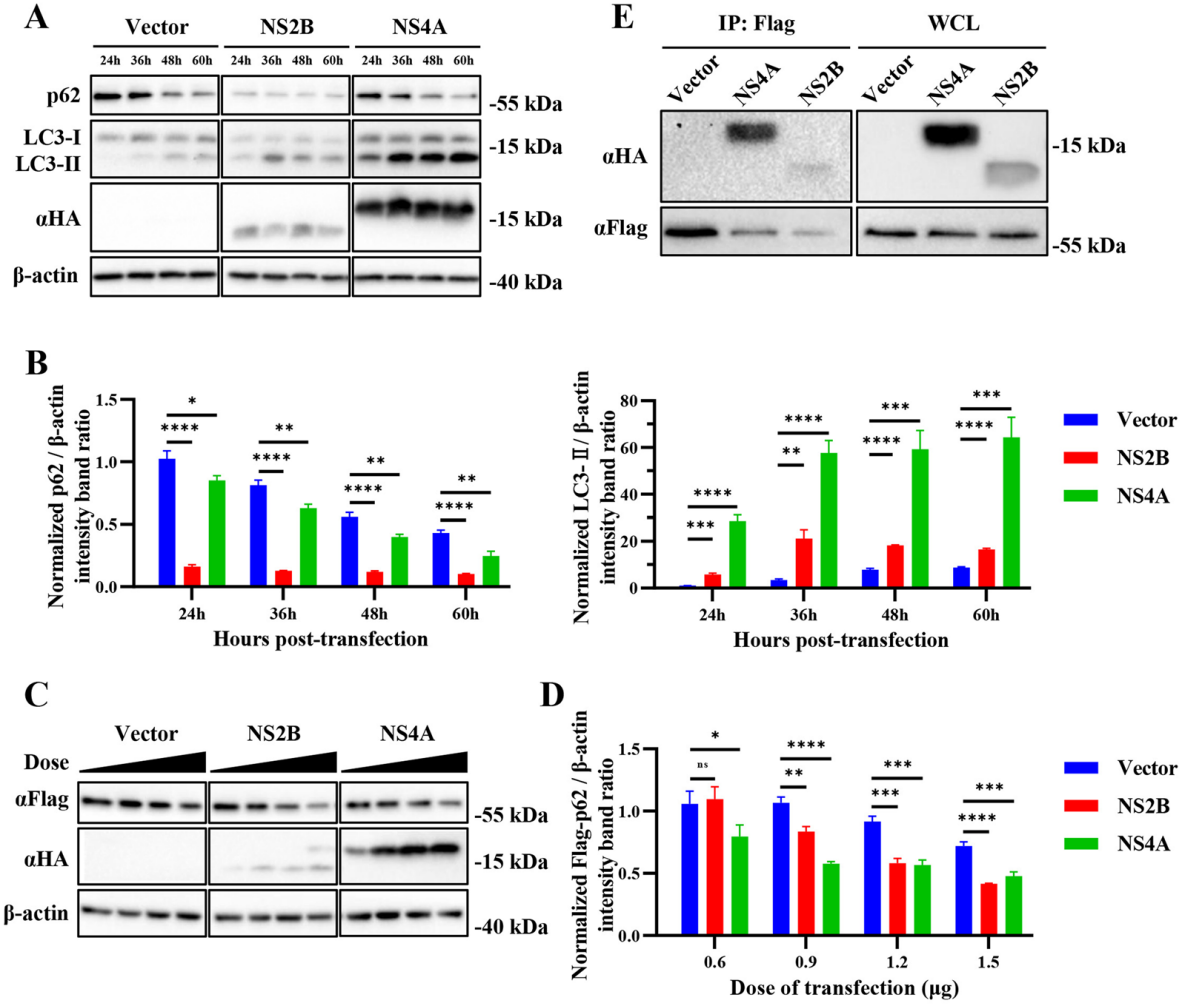
**NS2B and NS4A promote the degradation of p62. A** HEK293T cells were transfected with pCAGGS empty vector, pCAGGS-NS2B-HA, or pCAGGS-NS4A-HA for 24, 36, 48, and 60 h. Samples were harvested for Western blotting analysis and immunoblotted for the proteins p62, LC3, and β-actin. **B** Normalized p62/β-actin and LC3-II/β-actin intensity band ratios from the data in **A**. **C** HEK293T cells were co-transfected with pCAGGS-Flag-p62 and pCAGGS empty vector, pCAGGS-NS2B-HA, or pCAGGS-NS4A-HA for 36 h in a dose dependent manner (0.6, 0.9, 1.2, 1.5 µg). Samples were harvested for Western blotting analysis and immunoblotted for the proteins Flag, HA, and β-actin. **D** Normalized Flag-p62/β-actin intensity band ratio from the data in **C**. **E** HEK293T cells were co-transfected with pCAGGS-Flag-p62 and pCAGGS empty vector, pCAGGS-NS2B-HA, or pCAGGS-NS4A-HA for 36 h. Samples were harvested for Western blotting analysis and coimmunoprecipitation. Anti-Flag antibody which recognizes Flag-p62 was used to coimmunoprecipitate NS2B-HA and NS4A-HA. Student’s t test was performed to determine statistical significance (*, *P* < 0.05; **, *P* < 0.01; ***, *P* < 0.001; ****, *P* lt; 0.0001).

Since our previous work showed that NS2B and NS4A can promote autophagy, we next attempted to explore the effects of NS2B and NS4A on autophagic flux. First, NS2B and NS4A were overexpressed in HEK293T cells, and then, the production of LC3 and p62 was detected via Western blotting. As shown in Figures 4A and B and Additional files 2A and B, overexpression of NS2B and NS4A significantly enhanced the production of LC3-II and inhibited the production of p62 in a time-dependent manner, indicating that NS2B and NS4A enhanced both the initiation and degradation activities of autophagy. To further study the relationship between NS2B/NS4A and p62, HEK293T cells were co-transfected with Flag-tagged p62 and NS2B/NS4A. We found that the expression level of p62 was significantly inhibited by NS2B and NS4A in a dose-dependent manner (Figures 4C and D). Then we used coimmunoprecipitation to determine whether there was an interaction between p62 and NS2B/NS4A. As shown in Figure 4E and Additional file 2C, p62 interacted with NS2B and NS4A. These results suggest that NS2B and NS4A may interact with p62 to promote autophagic degradation activities, which results in the degradation of p62 and LC3-II.

### TMUV NS2B and NS4A enhance complete autophagy

**Figure 5:**
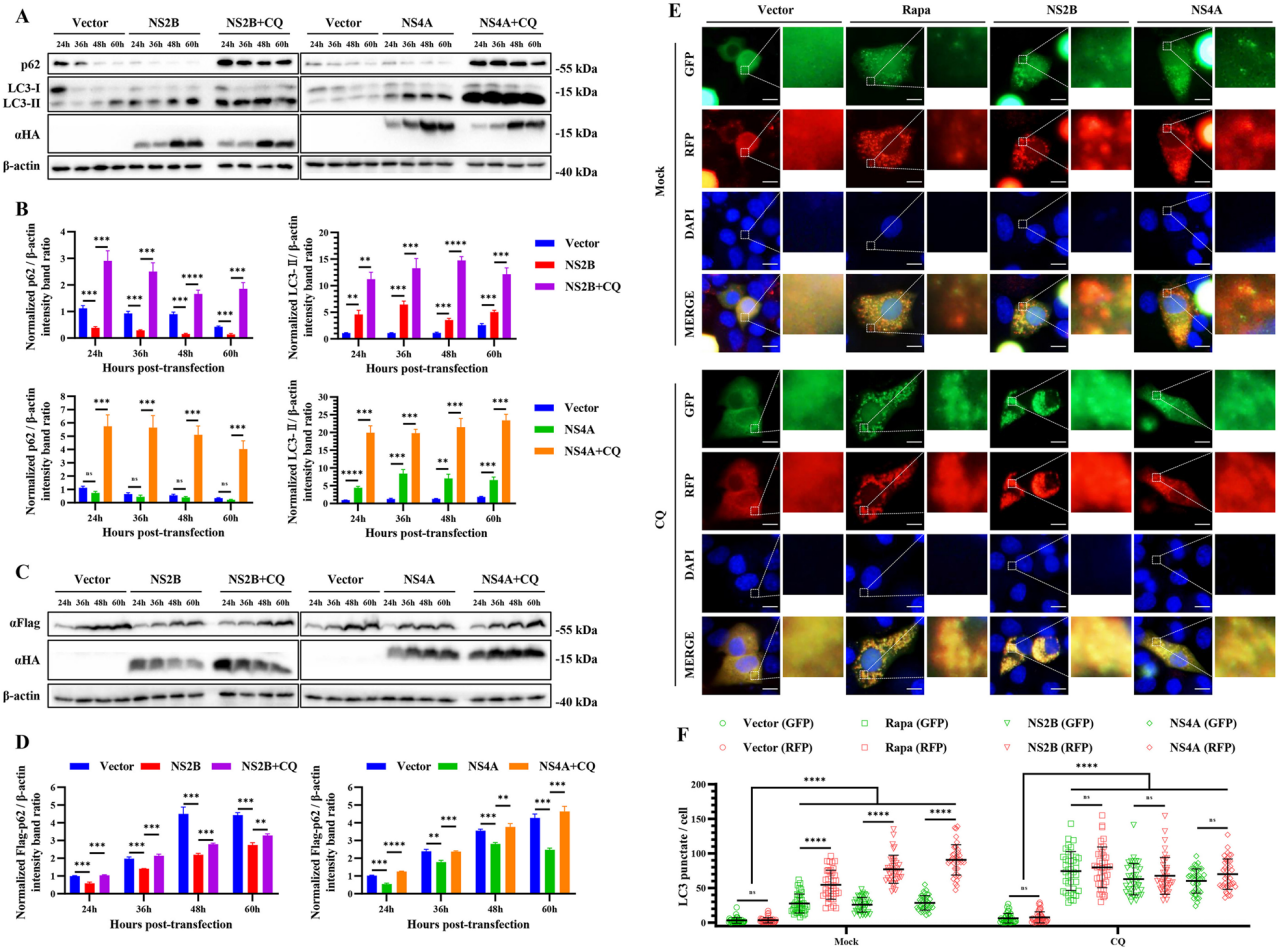
**TMUV NS2B and NS4A enhance complete autophagy**. **A** HEK293T cells treated with CQ (40 µM) or not were transfected with pCAGGS empty vector, pCAGGS-NS2B-HA, or pCAGGS-NS4A-HA for 24, 36, 48, and 60 h. Samples were harvested for Western blotting analysis and immunoblotted for the proteins p62, LC3, and β-actin. **B** Normalized p62/β-actin and LC3-II/β-actin intensity band ratios from the data in **A**. **C** HEK293T cells treated with CQ (40 µM) or not were co-transfected with pCAGGS-Flag-p62 and pCAGGS empty vector, pCAGGS-NS2B-HA, or pCAGGS-NS4A-HA for 24, 36, 48, and 60 h. Samples were harvested for Western blotting analysis and immunoblotted for the proteins Flag, HA, and β-actin. **D** Normalized Flag-p62/β-actin intensity band ratio from the data in **C**. **E** BHK-21 cells were co-transfected with ptf-LC3 and pCAGGS empty vector, pCAGGS-NS2B-HA, or pCAGGS-NS4A-HA. Cells co-transfected with ptf-LC3 and pCAGGS vector plasmids were used as negative controls, and cells treated with Rapa (200 nM) were used as positive controls. Cells were fixed and imaged for GFP and RFP fluorescence after transfection for 48 h. Bars, 10 μm. **F** Number of LC3 punctate fluorescence per cell. The LC3 puncta were counted in 40 cells per group. Student’s *t* test and one-way ANOVA were performed to determine statistical significance (*, *P* < 0.05; **, *P* < 0.01; ***, *P* < 0.001; ****, *P* < 0.0001).

The degradation of cargos during autophagic flux depends on the hydrolysis activity of proteases in the lysosome. To further verify that the degradation of LC3-II and p62 induced by NS2B and NS4A is due to the effect of autophagic flux, we utilized CQ to inhibit autophagic flux and observed whether the degradation of LC3-II and p62 could be reversed. As shown in Figures 5A-D, the endogenous expression of LC3-II and p62 was significantly reversed by CQ treatment, which was consistent with the exogenous expression of p62-Flag. After treatment with CQ, the production of p62 and LC3-II increased at least 6-fold and 2-fold respectively in NS2B transfected cells, and also increased at least 8-fold and 2-fold respectively in NS4A transfected cells (Figures 5A and B). These results demonstrate that NS2B and NS4A enhanced the autophagic degradation of LC3-II and p62. To further characterize these changes in the autophagic degradation of LC3-II, we performed an immunofluorescence assay in the presence or absence of CQ to identify autophagic flux. The results showed that the number of GFP-LC3 puncta per cell was significantly less than that of RFP-LC3 puncta in the absence of CQ (Figures 5E and F), suggesting that the expression of NS2B and NS4A promoted the degradation of GFP-LC3 puncta. However, the number of GFP-LC3 puncta per cell was reversed when cells were treated with CQ, and the overall fluorescence change in cells showed migration of RFP-LC3 (red) fluorescence to GFP-RFP-LC3 (yellow) fluorescence (Figures 5E and F), which suggested that the degradation of GFP-LC3 puncta was caused by autophagic flux. All our results suggest that TMUV NS2B and NS4A can enhance complete autophagy.

## Discussion

Autophagy is a lysosome-mediated catabolic process in which unwanted intracellular components are degraded to recycle nutrients for the regeneration of organelles and energy [9, 28]. Eukaryotic cells utilize autophagy to counteract stress responses, promote organelle turnover, eliminate aggregated proteins and excess lipids, degrade infectious microbes and regulate the immune response [9, 11, 29]. Therefore, autophagy protects cells from damage and maintains cellular homeostasis [30].

Flaviviruses have been widely considered serious pathogens that endanger public health [2]. An increasing number of studies have focused on the interaction between flaviviruses and autophagy [6, 31, 32]. The overall role of autophagy as proviral or antiviral in flavivirus replication is complex and has been less fully elucidated [6, 14]. Our previous study showed that autophagy could promote TMUV replication by suppressing p62-mediated innate immune responses in DEFs [12]. In this study, we verified that TMUV infection can induce autophagy and autophagy could promote the propagation of TMUV in HEK293T cells, which is consistent with our previous study.

New evidence has demonstrated that NS3 of TMUV can induce autophagy in DEFs [33]. However, our results showed that NS2B and NS4A are two key proteins involved in the initiation of autophagy. NS4A of flaviviruses is a small transmembrane protein that plays an important role in the viral life cycle and pathogenesis. NS4A mediates membrane remodeling induced by flaviviruses, participates in the formation of virus replication complexes, and interacts with host factors or other flavivirus NS proteins to promote efficient viral replication [26]. In addition, NS4A contributes to the pathogenesis of flaviviruses by counteracting the IFN response and modulating the UPR and autophagy through hijacking of a series of cellular signaling pathways [34–37]. For example, the cooperation of ZIKV NS4A and NS4B strongly suppresses host Akt-mTOR signaling, potentially leading to upregulation of autophagy and synergistically promoting viral replication [38]. DENV NS4A induces PI3K-dependent autophagy in epithelial cells and thus protected them from death to enhance viral infection [37]. DENV NS4A exploits the acyltransferase activity of AUP1 to trigger lipophagy (a selective autophagy) to promote viral production [20]. Consistent with previous studies, our results showed that NS4A of TMUV induces autophagy, which further confirms the important role of NS4A in the pathogenesis of flaviviruses. NS2B of flaviviruses forms a stable complex with NS3 and acts as a cofactor for the NS2B-NS3 serine protease, which is responsible for viral polyprotein processing [39]. Our previous study showed that binding of TMUV viral proteases to STING mediated by NS2B was crucial for STING cleavage and for impaired induction of IFN-β [40], suggesting an important role of NS2B in TMUV-host interactions. Given that NS2B must form a complex with NS3 to exert its serine protease activity, we also constructed a eukaryotic expression plasmid for NS2B3 (NS2B-NS3), which was overexpressed in HEK293T cells at the same time as other proteins. Interestingly, it was NS2B but not NS3 or NS2B3 that promoted the transition of LC3-I to LC3-II. These results suggest that TMUV NS2B has a novel function inducing autophagy, but this function of NS2B has not been reported in other flavivirus members.

Autophagy is a dynamic process involving the initiation and degradation of autophagosomes. Thus, it is reasonable to speculate that NS2B and NS4A can accelerate autophagic flux, also known as complete autophagy. In this study, we found that NS2B and NS4A interacted with p62 to degrade it and enhance autophagic flux, which is in agreement with our hypothesis. In addition, CQ inhibited NS2B- and NS4A-mediated degradation of p62 and GFP-LC3, providing additional strong evidence that NS2B and NS4A promote complete autophagy. Many studies have revealed that complete autophagy is required for the efficient replication of viruses, such as DENV, ZIKV and JEV [11, 41, 42]. As an emerging flavivirus, TMUV has been shown to induce complete autophagy to promote viral replication in DEFs [12], illustrating the important role of complete autophagy in the life cycle of TMUV. Our study highlighted the role of NS2B and NS4A in TMUV-induced complete autophagy, however, the mechanism by which NS2B and NS4A promote complete autophagy needs to be further explored.

Complete autophagy is accomplished by the recognition of microbial proteins or cargoes by autophagic receptors, such as p62 [43]. In p62-mediated autophagy, large cargoes such as aggregated proteins or cell organelles can be transported to lysosomes, in which p62 acts as a molecular linker [44]. Many studies have shown that p62 is involved in the host antiviral response [45–48]. For example, p62 recognizes and interacts with the M protein of SARS-CoV-2 during viral infection, promoting autophagic degradation of the M protein and thereby inhibiting viral propagation [46]. During infection with Seneca Valley virus (SVV), p62 targets SVV VP1 and VP3 to phagophores for degradation to inhibit viral replication [47]. Our recent study showed that p62 inhibits the propagation of TMUV by promoting innate immune responses in DEFs [12]. In the present study, we found that TMUV NS2B and NS4A interact with p62 to promote its autophagic degradation, which provides a possible explanation for the suppression of p62-mediated innate immune responses caused by TMUV infection.

Collectively, our study demonstrates that TMUV NS2B and NS4A are two crucial proteins that can induce complete autophagy to promote viral replication in HEK293T cells. Moreover, they can interact with the autophagy receptor p62 and promote its autophagic degradation. These results provide novel insight into the autophagy induced by TMUV, but the mechanism needs further investigation.

## Supplementary Information

**Additional file 1. Eukaryotic expression of viral proteins in HEK293T cells.** HEK293T cells were transfected with pCAGGS-C-HA, pCAGGS-prM-HA, pCAGGS-NS2A-HA and pCAGGS-NS4B-HA respectively for 48 h. Samples were harvested for Western blotting analysis and immunoblotted for the proteins HA. Bands labeled by “★” indicated the expressed specific viral proteins.

**Additional file 2. NS2B and NS4A can induce autophagy and interact with p62 in DEFs.** (A) DEFs were transfected with pCAGGS empty vector, pCAGGS-NS2B-HA, or pCAGGS-NS4A-HA for 24, 36, and 60 h. Samples were harvested for Western blotting analysis and immunoblotted for the proteins p62, LC3, and β-actin. (B) Normalized p62/β-actin and LC3-II/β-actin intensity band ratios from the data in (A). (C) DEFs were cotransfected with pCAGGS-Flag-p62 and pCAGGS empty vector, pCAGGS-NS2B-HA, or pCAGGS-NS4A-HA for 48 h. Samples were harvested for Western blotting analysis and coimmunoprecipitation. Anti-Flag antibody which recognizes Flag-p62 was used to coimmunoprecipitate NS2B-HA and NS4A-HA. Student’s *t* test was performed to determine statistical significance (*, *P* < 0.05; **, *P* < 0.01; ***, *P* < 0.001; ****, *P* < 0.0001).

## Data Availability

The datasets analyzed in this study are available from the corresponding author upon reasonable request.
