# Supplementary figures and images for: Nonstructural proteins 2B and 4A of Tembusu virus induce complete autophagy to promote viral multiplication in vitro
Source: Vet Res. 2023 Mar 14;54:23. doi: 10.1186/s13567-023-01152-2 (PMC10013240; doi:10.1186/s13567-023-01152-2)

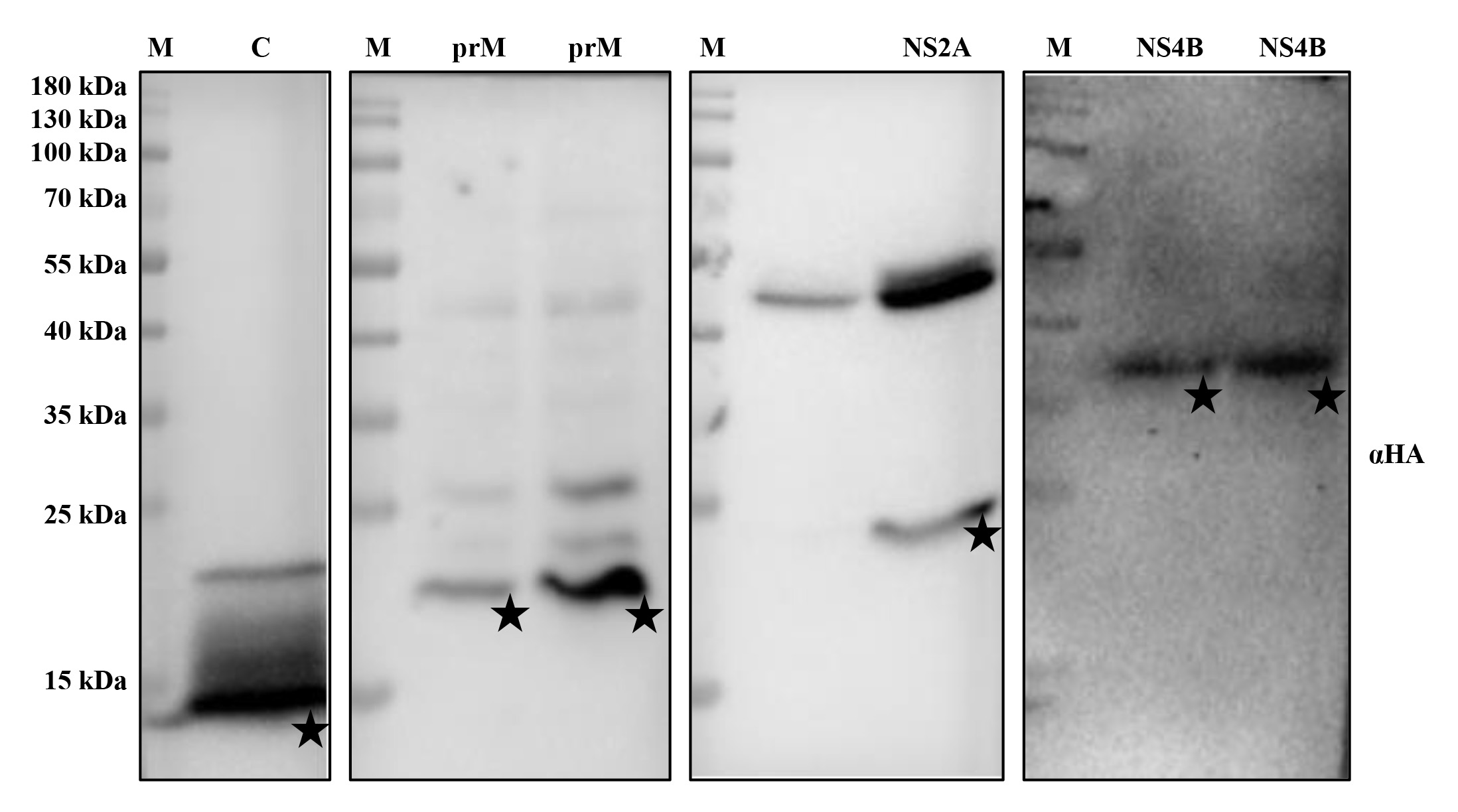

Supplement: Supplementary file 1 — Additional file 1. Eukaryotic expression of viral proteins in HEK293T cells. HEK293T cells were transfected with pCAGGS-C-HA, pCAGGS-prM-HA, pCAGGS-NS2A-HA and pCAGGS-NS4B-HA respectively for 48 h. Samples were harvested for Western blotting analysis and immunoblotted for the proteins HA. Bands labeled by “★” indicated the expressed specific viral proteins. [file 13567_2023_1152_MOESM1_ESM.jpg]

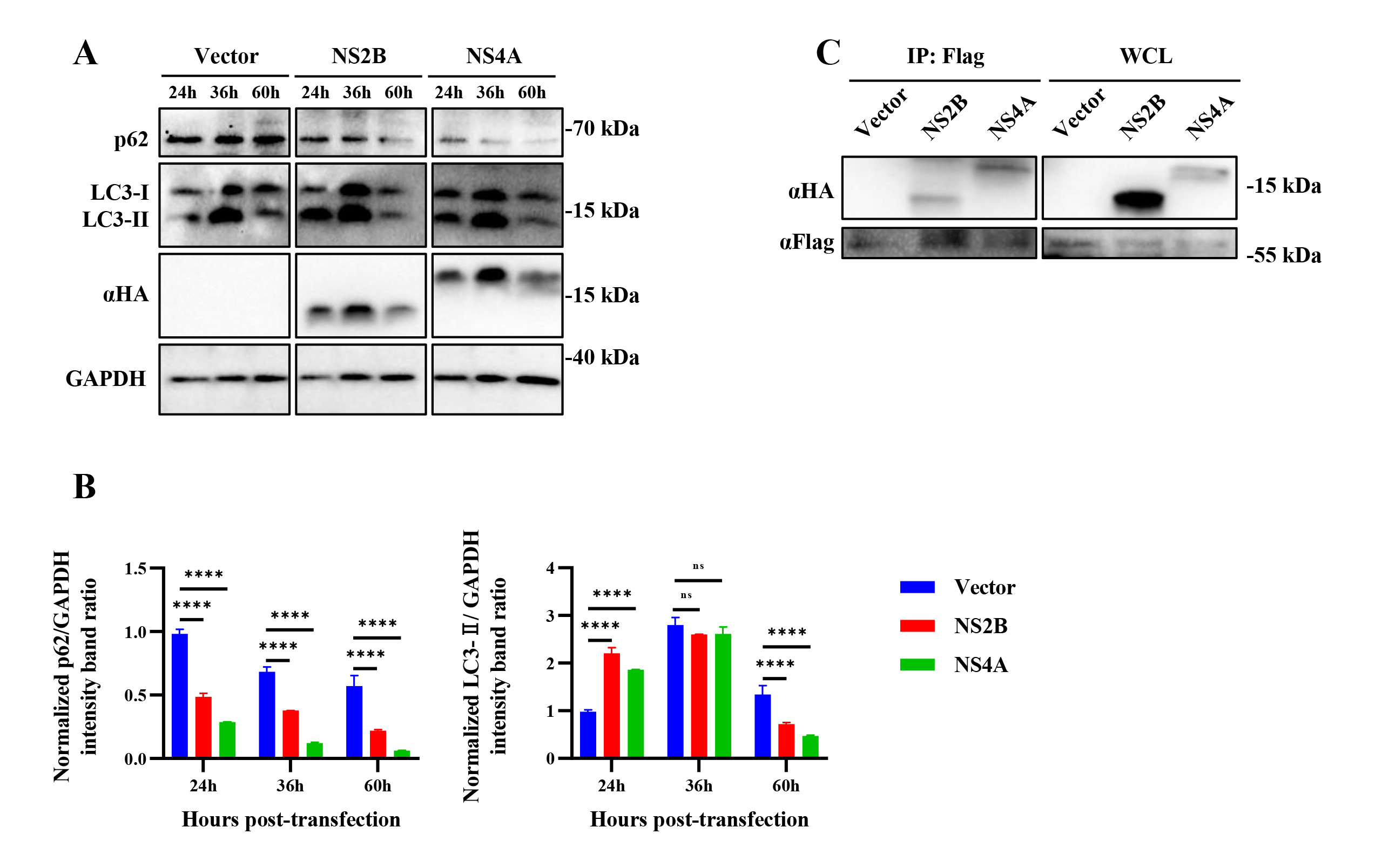

Supplement: Supplementary file 2 — Additional file 2. NS2B and NS4A can induce autophagy and interact with p62 in DEFs. (A) DEFs were transfected with pCAGGS empty vector, pCAGGS-NS2B-HA, or pCAGGS-NS4A-HA for 24, 36, and 60 h. Samples were harvested for Western blotting analysis and immunoblotted for the proteins p62, LC3, and β-actin. (B) Normalized p62/β-actin and LC3-II/β-actin intensity band ratios from the data in (A). (C) DEFs were cotransfected with pCAGGS-Flag-p62 and pCAGGS empty vector, pCAGGS-NS2B-HA, or pCAGGS-NS4A-HA for 48 h. Samples were harvested for Western blotting analysis and coimmunoprecipitation. Anti-Flag antibody which recognizes Flag-p62 was used to coimmunoprecipitate NS2B-HA and NS4A-HA. Student’s t test was performed to determine statistical significance (*, P < 0.05; **, P < 0.01; ***, P < 0.001; ****, P < 0.0001). [file 13567_2023_1152_MOESM2_ESM.jpg]
